# Translating drug-resistant tuberculosis surveillance into molecular–biophotonic diagnostic innovation in rural Eastern Cape, South Africa

**DOI:** 10.3389/ftubr.2026.1937755

**Published:** 2026-10-12

**Authors:** Ncomeka Sineke, Kamvelihle Sabisa, Ntandazo Dlatu, Saturnin Ombinda-Lemboumba, Teke Apalata, Lindiwe Modest Faye

**Affiliations:** 1 WSU—TB Research Group, School of Pathology, Faculty of Medicine and Health Sciences, Walter Sisulu University, Mthatha, South Africa; 2 Walter Sisulu Institute for Clinical Governance, Healthcare Administration, School of Public Health, Faculty of Medicine and Health Sciences, Walter Sisulu University, Mthatha, South Africa; 3 Biophotonics, Photonic Centre, Manufacturing Cluster, Council for Scientific and Industrial Research (CSIR), Pretoria, South Africa

**Keywords:** biophotonics, biosensors, drug-resistant tuberculosis, epidemiological surveillance, molecular diagnostics, molecular target prioritization, mycobacterium tuberculosis, point-of-care testing

## Abstract

**Background:**

Drug-resistant tuberculosis (DR-TB) remains a major public health challenge in South Africa, particularly in rural settings with constrained access to rapid molecular diagnostics. There is a need to identify locally relevant molecular targets that could inform the future development of affordable point-of-care (POC) diagnostic platforms. This study used routine DR-TB surveillance data to develop an epidemiology-guided framework for prioritizing molecular targets for future molecular–biophotonic diagnostic development.

**Methods:**

A retrospective secondary analysis was conducted using routine DR-TB programme data from 402 patients managed at two decentralized treatment facilities in the rural Eastern Cape Province, South Africa, between 2020 and 2024. Resistance categories, treatment outcomes, and programme vulnerability indicators were analysed descriptively. A diagnostic prioritization framework integrating disease burden, frequency of resistance categories, treatment outcome vulnerability, programme impact, and potential suitability for decentralized implementation was used to prioritize established resistance-associated molecular targets. Molecular targets were informed by published evidence; no isolate-level mutation analysis, sequencing, biosensor development, or analytical or clinical validation of a diagnostic platform was performed.

**Results:**

Rifampicin-resistant tuberculosis (RR-TB) (47.5%) and multidrug-resistant tuberculosis (MDR-TB) (44.3%) accounted for 91.8% of DR-TB cases, while pre-extensively drug-resistant tuberculosis (pre-XDR-TB) and extensively drug-resistant tuberculosis (XDR-TB) accounted for 5.7% and 2.5%, respectively. Declining treatment success and increasing proportions of unevaluated outcomes among RR-TB and MDR-TB indicated programme vulnerability; however, the available data did not permit causal attribution of these patterns to diagnostic limitations. The framework prioritized *rpoB, katG,* and *inhA* as high-priority targets, followed by *gyrA, gyrB, rrs,* and *eis* as medium-priority targets, with *embB, pncA, Rv0678, atpE,* and *pepQ* identified for future expansion. These targets were conceptually mapped to candidate biophotonic biosensing approaches; their diagnostic performance and POC suitability were not evaluated in this study.

**Conclusions:**

This study proposes an epidemiology-guided framework for prioritizing molecular targets for future DR-TB diagnostic development in rural settings. The molecular–biophotonic component is conceptual and does not represent a validated diagnostic technology. Mutation-level characterization, biosensor development, analytical evaluation, and prospective clinical validation are required to determine the performance, feasibility, and potential POC applicability of the proposed approach.

## 1 Introduction

Tuberculosis (TB) remains a leading infectious cause of morbidity and mortality worldwide despite substantial advances in diagnosis, treatment, and disease control (1). According to the World Health Organization (WHO), approximately 10.8 million people developed TB and 1.25 million died from the disease in 2023, with drug-resistant tuberculosis (DR-TB) continuing to threaten progress towards the End TB Strategy (2, 3). Rifampicin-resistant tuberculosis (RR-TB), multidrug-resistant tuberculosis (MDR-TB), pre-extensively drug-resistant tuberculosis (pre-XDR-TB), and extensively drug-resistant tuberculosis (XDR-TB) are associated with complex treatment, increased healthcare costs, adverse clinical outcomes, and continued transmission (4). Rapid and accurate identification of drug resistance is therefore important for guiding appropriate treatment and strengthening DR-TB control (5). South Africa remains a high-burden TB and DR-TB setting, with substantial challenges in rural areas of the Eastern Cape Province (6). Although molecular technologies such as Xpert MTB/RIF Ultra and line probe assays have improved the detection of TB and drug resistance, access to rapid molecular testing may remain constrained in some rural settings by laboratory capacity, trained personnel, specimen transportation, infrastructure, and equipment requirements (7, 8). These health-system constraints support continued investigation of decentralized diagnostic approaches that could complement existing laboratory-based services (1, 9). Point-of-care (POC) diagnostic technologies have consequently received increasing attention as potential approaches for expanding access to rapid testing in resource-constrained settings (10). Molecular diagnostics detect resistance-associated genetic variants, while emerging biophotonic approaches use optical principles, including fluorescence, surface plasmon resonance (SPR), localized surface plasmon resonance (LSPR), photonic crystal sensing, and surface-enhanced Raman spectroscopy (SERS), for nucleic acid or biomolecular detection (11, 12). Published studies suggest that some of these technologies may offer opportunities for miniaturization and multiplex assay development. However, their analytical performance, portability, operational feasibility, and clinical suitability depend on the specific platform and require experimental validation before POC implementation. Drug resistance in *Mycobacterium tuberculosis* (*M. tuberculosis*) is associated with genetic variation in several established resistance-related loci. Rifampicin resistance is predominantly associated with mutations in *rpoB*, while isoniazid resistance commonly involves *katG* and the *inhA* promoter region (13). Resistance to other anti-TB drugs is associated with additional targets, including *gyrA, gyrB, rrs, eis, embB, pncA, Rv0678, atpE,* and *pepQ* (14, 15). These established molecular targets provide potential candidates for future multiplex diagnostic development. In resource-constrained settings, prioritizing targets according to locally relevant resistance patterns may help guide phased diagnostic development where initial multiplex capacity is limited. Routine surveillance data from the rural Eastern Cape indicate that RR-TB and MDR-TB constitute a substantial proportion of DR-TB managed within decentralized treatment programmes (6, 16). Programme data have also documented changes in treatment success and the proportion of patients with unevaluated outcomes (17). These patterns should not, however, be interpreted as evidence that diagnostic limitations caused poorer treatment outcomes. DR-TB outcomes are multifactorial and may be influenced by clinical characteristics, HIV status, treatment regimens, adherence, loss to follow-up, healthcare access, socioeconomic circumstances, and other health-system factors. Nevertheless, surveillance patterns can help identify areas of programme vulnerability and inform priorities for further diagnostic research. Secondary analysis of routine programme data may therefore provide a pragmatic approach for informing early-stage diagnostic prioritization (18, 19). Surveillance data can identify locally prevalent resistance categories and programme vulnerabilities, while established molecular evidence can inform the selection of corresponding resistance-associated targets (20). Integrating these sources of evidence may provide a structured approach for identifying molecular targets for subsequent laboratory development. Importantly, such prioritization does not demonstrate the presence or frequency of specific mutations in the study population unless supported by isolate-level molecular analysis.

This study, therefore, used routine DR-TB surveillance data from the rural Eastern Cape to develop an epidemiology-guided framework for prioritizing molecular targets for future molecular–biophotonic diagnostic development. The framework integrates the observed distribution of DR-TB resistance categories and programme-level indicators with established resistance-associated molecular targets reported in the literature. The study does not analyse mutation frequencies at the isolate level, develop a biosensor, or evaluate the analytical or clinical performance of a diagnostic technology. Rather, it proposes a hypothesis-generating translational framework to guide subsequent mutation-level characterization, biosensor development, analytical evaluation, and prospective clinical validation.

## 2 Materials and methods

### 2.1 Study design

This study employed a retrospective secondary analysis of routinely collected drug-resistant tuberculosis (DR-TB) programme data from two decentralized treatment facilities in the Eastern Cape Province, South Africa. The analysis aimed to characterize the distribution of DR-TB resistance categories and treatment outcomes and to use these data to develop an epidemiology-guided framework for prioritizing molecular targets for future diagnostic development. The study did not evaluate treatment effectiveness, analyse isolate-level mutations, develop a biosensor, or assess the analytical or clinical performance of a diagnostic technology.

### 2.2 Study setting

The study used data from two decentralized DR-TB treatment facilities serving rural communities in the Eastern Cape Province: Mthatha Gateway Clinic and Holy Cross Clinic. The participating facilities provide treatment initiation, monitoring, and follow-up for patients with rifampicin-resistant tuberculosis (RR-TB), multidrug-resistant tuberculosis (MDR-TB), pre-extensively drug-resistant tuberculosis (pre-XDR-TB), and extensively drug-resistant tuberculosis (XDR-TB) within South Africa's decentralized DR-TB programme.

The Eastern Cape includes geographically dispersed rural communities where access to specialized diagnostic and treatment services may be affected by health-system and geographic constraints. These characteristics provided an appropriate context for examining DR-TB programme patterns and considering priorities for future decentralized diagnostic development.

### 2.3 Data sources

The secondary dataset was derived from routinely collected DR-TB programme data that had previously undergone quality assurance and validation. Data were obtained from the Electronic Drug-Resistant Tuberculosis Register (EDRWeb), facility-based DR-TB treatment registers, district TB reports, and supporting laboratory records. Cross-validation across these sources had previously been undertaken to improve data completeness and identify duplicate treatment episodes.

The present study used the validated dataset without additional patient recruitment, specimen collection, laboratory testing, or molecular sequencing.

### 2.4 Study population and resistance classification

The study included patients with bacteriologically confirmed DR-TB who initiated treatment between January 2020 and December 2024. Patients without documented treatment initiation or with incomplete resistance classification were excluded from the original dataset and were therefore not included in this secondary analysis.

Resistance categories were defined according to established definitions (21):.

- **RR-TB:** TB caused by *M. tuberculosis* resistant to rifampicin, with or without resistance to other drugs.
- **MDR-TB:** TB caused by *M. tuberculosis* resistant to at least rifampicin and isoniazid.
- **Pre-XDR-TB:** MDR/RR-TB with additional resistance to any fluoroquinolone.
- **XDR-TB:** MDR/RR-TB with additional resistance to any fluoroquinolone and at least one other WHO Group A drug, specifically bedaquiline or linezolid.

The dataset contained resistance-category classifications but did not include isolate-level sequencing results, mutation frequencies, or individual genotype–phenotype associations. Consequently, the molecular determinants associated with these resistance categories were not directly measured in the study population.

### 2.5 Epidemiology-guided diagnostic prioritization framework

A conceptual Translational Molecular–Biophotonic Diagnostic Framework was developed to link routine DR-TB surveillance findings with molecular-target prioritization for future diagnostic research (Figure 1). The framework comprised four stages: (1) characterization of the distribution of DR-TB resistance categories; (2) description of programme-level treatment outcomes; (3) prioritization of established resistance-associated molecular targets; and (4) conceptual mapping of prioritized targets to candidate molecular–biophotonic technologies.

**Figure 1:**
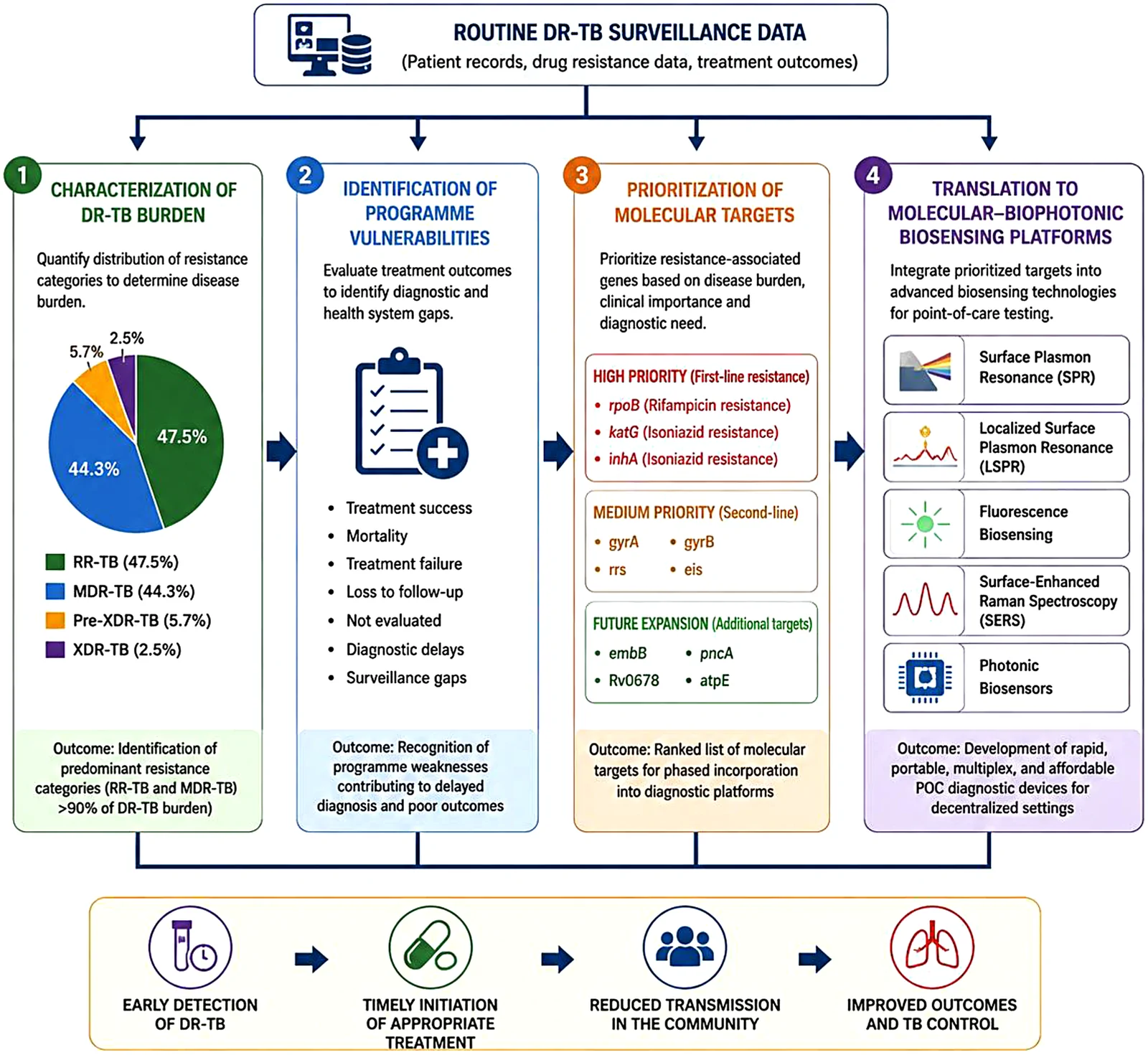
Translational molecular–Biophotonic diagnostic framework for point-of-care detection of drug-resistant *M. tuberculosis*. The framework illustrates the proposed pathway from routine DR-TB surveillance to future diagnostic development. Surveillance data are used to characterize resistance-category burden and programme-level outcomes, followed by prioritization of established resistance-associated molecular targets and conceptual mapping to candidate molecular–biophotonic technologies. No isolate-level mutation analysis, biosensor development, or analytical or clinical validation was undertaken in this study.

**Figure 2:**
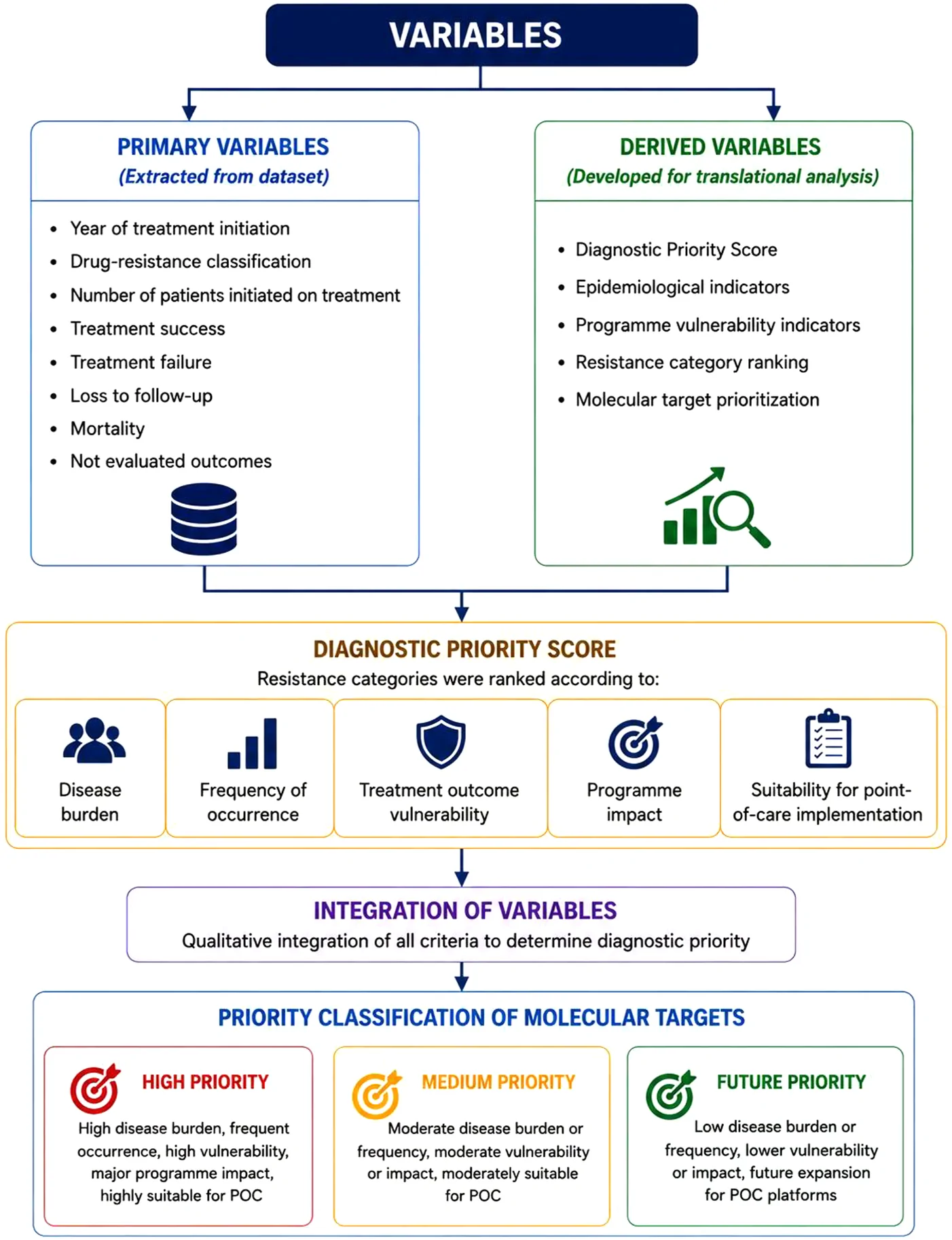
Variable selection and diagnostic priority framework for molecular target prioritization. This flowchart illustrates the process used to translate routine DR-TB surveillance data into molecular-target priorities for future diagnostic development. Primary variables included year of treatment initiation, drug-resistance classification, number of patients initiated on treatment, treatment success, treatment failure, loss to follow-up, mortality, and unevaluated outcomes. Resistance categories were assessed using five predefined domains: disease burden, frequency of occurrence, treatment outcome vulnerability, programme impact, and potential suitability for decentralized diagnostic development. These criteria informed classification into high-, medium-, and future-priority categories. Molecular targets were subsequently mapped to resistance categories using established evidence from the molecular TB literature. The framework represents a conceptual prioritization process; no isolate-level mutation analysis or diagnostic technology validation was undertaken. Following the epidemiological analysis, each drug-resistance category was mapped to established molecular determinants of *M. tuberculosis* drug resistance reported in the literature. The dataset contained resistance-category classifications but did not include gene-level sequencing results, targeted mutation profiles, mutation frequencies, or individual genotype–phenotype associations. Consequently, molecular targets were not identified from mutations measured directly in the study population. Molecular-target prioritization considered epidemiological burden, clinical relevance, and potential diagnostic feasibility. Epidemiological importance was based primarily on the proportional contribution of each resistance category to the observed DR-TB caseload. Clinical relevance considered the implications of the corresponding resistance category for treatment selection, while diagnostic feasibility considered the established molecular relevance of the targets and their potential suitability for future molecular detection. These criteria were used for conceptual prioritization and were not intended as validated measures of diagnostic performance. Based on this framework, resistance-associated molecular targets were grouped as presented in Table 1.

**Table 1:** Epidemiology-guided prioritization of molecular targets for future drug-resistant TB diagnostic development.

Priority	Molecular targets	Principal resistance relevance
High	*rpoB, katG, inhA*	Rifampicin and isoniazid resistance
Medium	*gyrA, gyrB, rrs, eis*	Fluoroquinolone and selected second-line drug resistance
Future expansion	*embB, pncA, Rv0678, atpE, pepQ*	Expanded drug-resistance detection

The framework was intended as a hypothesis-generating prioritization approach rather than a validated diagnostic model. Programme outcome patterns were used to characterize programme vulnerability and were not interpreted as evidence that diagnostic limitations caused poor treatment outcomes. Similarly, the mapping of molecular targets to candidate biophotonic technologies was conceptual and did not constitute evidence of analytical or clinical performance.

### 2.6 Variables and diagnostic priority classification

Variables extracted from the surveillance dataset included year of treatment initiation, drug-resistance classification, number of patients initiated on treatment, treatment success, treatment failure, loss to follow-up, mortality, and unevaluated outcomes. These variables were used to describe the distribution of DR-TB resistance categories and programme-level treatment outcomes. An exploratory Diagnostic Priority Index (DPI) was developed to translate these descriptive findings into molecular-target priorities. The DPI considered five domains: disease burden, frequency of resistance categories, treatment outcome vulnerability, programme impact, and potential suitability for decentralized diagnostic development. For pragmatic prioritization, resistance categories accounting for ≥40% of the observed DR-TB caseload were considered high epidemiological priority; those accounting for 5%–39% were considered medium priority; and those accounting for <5% were considered for future expansion when clinically relevant. These thresholds were predefined decision boundaries within the conceptual framework and were not statistically or clinically validated cutoffs. Treatment outcomes were considered contextual indicators of programme vulnerability rather than determinants of diagnostic priority in a causal sense. No causal relationship between diagnostic limitations and treatment outcomes was assumed or tested.

The prioritization reflects the combination of the observed distribution of resistance categories and the established molecular relevance of the corresponding targets. It should not be interpreted as representing mutation frequencies measured in the study population.

### 2.7 Conceptual mapping to molecular–biophotonic technologies

The prioritized molecular targets were conceptually mapped to candidate molecular–biophotonic biosensing technologies reported in the literature. Candidate approaches included surface plasmon resonance (SPR), localized surface plasmon resonance (LSPR), fluorescence-based biosensing, surface-enhanced Raman spectroscopy (SERS), photonic crystal biosensors, and integrated optical waveguide biosensors. The literature-informed mapping considered characteristics potentially relevant to decentralized diagnostic development, including multiplex capability, analytical sensitivity, turnaround time, portability, infrastructure requirements, and potential suitability for point-of-care implementation. These characteristics were not measured experimentally in the present study and therefore represent potential technological attributes rather than study findings. No biosensor was fabricated or experimentally tested, and no analytical sensitivity, specificity, limit of detection, reproducibility, multiplex performance, portability, diagnostic accuracy, or clinical feasibility was evaluated. Accordingly, the molecular–biophotonic component represents a conceptual, hypothesis-generating framework for guiding future technology development. Laboratory development, analytical evaluation, and prospective clinical validation would be required to determine the performance and potential point-of-care applicability of any resulting diagnostic platform.

### 2.8 Statistical analysis

Descriptive statistical analyses were performed to characterize the distribution of DR-TB resistance categories and treatment outcomes between 2020 and 2024. Categorical variables were summarized as frequencies and percentages. The proportional contribution of each resistance category to the overall DR-TB caseload was calculated, and treatment outcomes were summarized by resistance category and year of treatment initiation. Where applicable, previously estimated proportions were presented with 95% Wilson confidence intervals.

Programme vulnerability was assessed descriptively using treatment success, mortality, treatment failure, loss to follow-up, and unevaluated outcomes. These indicators were used to describe programme-level patterns and were not interpreted as evidence that diagnostic limitations caused the observed treatment outcomes.

No inferential statistical analyses were performed. Specifically, no hypothesis tests, regression models, formal tests for temporal trends, or between-group significance tests were undertaken. Year-specific treatment-outcome patterns were therefore interpreted descriptively, particularly where resistance categories had few observations. The descriptive findings informed the epidemiology-guided prioritization framework but were not used to establish causal relationships between diagnostic access, diagnostic delay, treatment characteristics, and treatment outcomes. Statistical analyses were conducted using IBM SPSS Statistics version 29.0 (IBM Corp., Armonk, NY, USA) and R version 4.3.2 (R Foundation for Statistical Computing, Vienna, Austria).

## 3 Results

### 3.1 Distribution of drug-resistant TB categories

A total of 402 patients with DR-TB were included in the secondary analysis. RR-TB was the most prevalent resistance category, accounting for 191 (47.5%) patients, followed by MDR-TB with 178 (44.3%) patients. Collectively, RR-TB and MDR-TB accounted for 369 of 402 cases (91.8%), representing most of the DR-TB burden in the study population. In comparison, pre-XDR-TB accounted for 23 (5.7%) cases, while XDR-TB represented the smallest subgroup with 10 (2.5%) cases (Table 2). The marked predominance of RR-TB and MDR-TB over pre-XDR-TB and XDR-TB demonstrates that resistance to first-line anti-TB drugs constituted the principal form of drug resistance observed during the study period. These epidemiological patterns formed the basis for the subsequent molecular target prioritization analysis.

**Table 2:** Distribution of drug-resistant TB categories.

Resistance category	Number (*n*)	Percentage (%)
RR-TB	191	47.5
MDR-TB	178	44.3
Pre-XDR-TB	23	5.7
XDR-TB	10	2.5
**Total**	**402**	**100**

Bold values indicate the total number of the distribution of drug-resistant categories and the percentage.

### 3.2 Epidemiology-guided prioritization of molecular targets

Application of the predefined evidence informed DPI identified distinct priority levels for resistance-associated molecular targets based on epidemiological burden, programme vulnerability, clinical significance, and diagnostic feasibility. Resistance categories associated with RR-TB and MDR-TB had the highest diagnostic priority because they collectively accounted for 91.8% (47.5% and 44.3% respectively) of all DR-TB cases in the study population. Consequently, the corresponding resistance-associated genes, *rpoB*, *katG*, and *inhA*, were classified as high-priority molecular targets for point-of-care diagnostic development (Table 3). Genes associated with second-line drug resistance were assigned a lower priority based on the relative frequency of the corresponding resistance categories. Mutations in *gyrA*, *gyrB*, *rrs*, and *eis*, which are associated with fluoroquinolone and injectable drug resistance, were classified as medium-priority targets because pre-XDR-TB represented only 5.7% of the study population. Additional resistance-associated genes, including *embB*, *pncA*, *Rv0678*, *atpE*, and *pepQ*, were categorized as future-priority targets, reflecting the comparatively low prevalence of XDR-TB (2.5%) and the phased molecular target prioritization framework developed in this study. The resulting priority classification established a three-tier molecular target framework comprising high-, medium-, and future-priority categories, providing the basis for the subsequent conceptual translation of prioritized molecular targets into molecular–biophotonic point-of-care diagnostic design, however, mutation frequencies were not measured in the present dataset.

**Table 3:** Epidemiology-guided prioritization of resistance-associated molecular targets for molecular–biophotonic point-of-care diagnosis of drug-resistant Mtb.

Resistance/diagnostic domain	Estimated priority	Principal molecular targets	Diagnostic priority
RR-TB	Very high	*rpoB*	High
MDR-TB	Very high	*rpoB, katG, inhA*	High
Fluoroquinolone resistance/pre-XDR-TB	Moderate	*gyrA, gyrB*	Medium
Additional second-line drug resistance	Moderate	*rrs, eis*	Medium
Expanded resistance detection	Future expansion	*embB, pncA, Rv0678, atpE, pepQ*	Future

### 3.3 Programme vulnerability and diagnostic need

Analysis of treatment outcome trends demonstrated increasing programme vulnerability among the two predominant resistance categories, RR-TB and MDR-TB. Among patients with MDR-TB, treatment success declined from 80.9% in 2021 to 38.5% in 2024, while the proportion of patients classified as not evaluated increased from 0% to 30.8% over the same period. Similarly, treatment success among patients with RR-TB decreased from 79.5% to 51.6%, accompanied by an increase in not evaluated outcomes to 25.8%. By comparison, pre-XDR-TB and XDR-TB accounted for only 5.7% and 2.5% of the total DR-TB burden, respectively. Owing to the relatively small number of cases within these resistance categories, temporal treatment outcome patterns were less stable and were therefore interpreted descriptively. Nevertheless, these findings indicate that programme vulnerability was most evident among the resistance categories contributing the greatest proportion of the overall DR-TB burden and formed an integral component of the DPI used for molecular target prioritization.

### 3.4 Diagnostic priority matrix

Application of the Diagnostic Priority Index generated a three-tier priority matrix for DR-TB resistance categories based on their epidemiological burden, programme vulnerability, clinical significance, and diagnostic feasibility (Table 4). RR-TB and MDR-TB were assigned the highest overall priority rankings because they represented the largest proportion of the DR-TB burden and demonstrated the greatest programme vulnerability, characterized by declining treatment success and increasing proportions of unevaluated treatment outcomes. Both resistance categories also received the highest ratings for public health impact owing to their predominance within the study population. In contrast, pre-XDR-TB and XDR-TB were assigned lower overall priority rankings. Although these resistance categories were of substantial clinical importance, their comparatively low prevalence resulted in lower epidemiological priority scores within the DPI. Based on the integrated assessment, RR-TB and MDR-TB were classified as Priority 1 targets for molecular–biophotonic diagnostic development, pre-XDR-TB as Priority 2, and XDR-TB as Priority 3 (Table 4).

**Table 4:** Diagnostic priority matrix for epidemiology-guided prioritization of DR-TB categories for molecular–Biophotonic point-of-care diagnostic development.

Criterion	RR-TB	MDR-TB	Pre-XDR-TB	XDR-TB
Disease burden	★★★★★	★★★★★	★★⋆⋆⋆	★⋆⋆⋆⋆
Programme vulnerability	★★★★⋆	★★★★★	★★⋆⋆⋆	★★★⋆⋆
Public health impact	★★★★★	★★★★★	★★★⋆⋆	★★★★⋆
**Overall diagnostic priority**	**Priority 1**	**Priority 1**	**Priority 2**	**Priority 3**

### 3.5 Prioritized molecular target panel

Application of the Diagnostic Priority Index resulted in a phased molecular target panel comprising three levels of diagnostic priority (Table 5). The high-priority panel included *rpoB*, *katG*, and *inhA*, corresponding to rifampicin- and isoniazid-resistant TB, which collectively represented the predominant drug-resistance categories identified in the study population. These targets were assigned the highest priority based on their combined epidemiological burden, programme vulnerability, clinical significance, and diagnostic feasibility. The medium-priority panel comprised *gyrA*, *gyrB*, *rrs*, and *eis*, representing molecular determinants associated with resistance to fluoroquinolones and injectable drugs. The future-priority panel included *embB*, *pncA*, *Rv0678*, *atpE* and *pepQ* which are associated with resistance to additional first- and second-line anti-TB drugs. Collectively, the phased molecular target panel provides an epidemiology-guided framework for the progressive incorporation of resistance-associated genes into molecular–biophotonic diagnostic platforms.

**Table 5:** Phased molecular target panel prioritized for molecular–Biophotonic point-of-care diagnosis of drug-resistant mtb.

Priority level	Molecular targets	Associated drug resistance
High	*rpoB*, *katG*, *inhA*	Rifampicin and isoniazid resistance
Medium	*gyrA*, *gyrB*, *rrs*, *eis*	Fluoroquinolone and injectable drug resistance
Future	*embB*, *pncA*, *Rv0678*, *atpE, pepQ*	Expanded resistance to first- and second-line anti-TB drugs

### 3.6 Compatibility of prioritized molecular targets with molecular–biophotonic biosensing technologies

The prioritized molecular targets identified through the DPI were conceptually evaluated for compatibility with emerging molecular–biophotonic diagnostic technologies. The compatibility assessment demonstrated that the high-priority molecular targets (*rpoB*, *katG*, and *inhA*) are potentially compatible with multiple optical biosensing platforms capable of nucleic acid-based mutation detection under decentralized testing conditions. These targets exhibited favourable characteristics for integration into biophotonic diagnostic systems owing to their well-characterized resistance-associated mutations and compatibility with molecular amplification strategies. Among the technologies evaluated, SPR, LSPR, and fluorescence-based biosensing demonstrated potential compatibility with all high-priority molecular targets, supporting rapid, multiplex molecular detection. These platforms were characterized by high analytical sensitivity, label-free or minimally labelled detection, multiplex detection capability, rapid analytical turnaround, miniaturization potential, and compatibility with portable point-of-care instrumentation (Table 6). The compatibility assessment further demonstrated that medium- and future-priority molecular targets, including *gyrA*, *gyrB*, *rrs*, *eis*, *embB*, *pncA*, *Rv0678*, *atpE* and *pepQ*, could be incorporated into the same conceptual molecular–biophotonic framework, supporting a phased expansion of multiplex diagnostic capability.

**Table 6:** Conceptual compatibility of prioritized molecular targets with molecular–Biophotonic biosensing technologies.

Biophotonic technology	High-priority targets	Multiplex capability	Portable POC suitability
Surface plasmon resonance (SPR)	*rpoB*, *katG*, *inhA*	High	High
Localized surface plasmon resonance (LSPR)	*rpoB*, *katG*, *inhA*	High	High
Fluorescence-based biosensing	*rpoB*, *katG*, *inhA*	High	High
Surface-enhanced Raman spectroscopy (SERS)	High- and medium-priority targets	Moderate–High	Moderate
Photonic crystal biosensors	High- and medium-priority targets	High	High
Integrated optical waveguide biosensors	High-, medium-, and future-priority targets		

Collectively, the epidemiology-guided prioritization framework identified RR-TB and MDR-TB as the predominant DR-TB categories, accounting for 91.8% of the total DR-TB burden in the study population. Application of the Diagnostic Priority Index classified *rpoB*, *katG*, and *inhA* as high-priority molecular targets, while *gyrA*, *gyrB*, *rrs*, and *eis* were classified as medium-priority, and *embB*, *pncA*, *Rv0678*, *atpE* and *pepQ* as future-priority targets. The conceptual compatibility assessment demonstrated that these prioritized molecular targets can be aligned with multiple molecular–biophotonic biosensing technologies, providing an epidemiology-guided framework for phased diagnostic platform development.

## 4 Discussion

This study used routine DR-TB surveillance data to develop an epidemiology-guided framework for prioritizing molecular targets for future molecular–biophotonic diagnostic development. RR-TB and MDR-TB accounted for 91.8% of the observed DR-TB burden, supporting the prioritization of *rpoB, katG,* and *inhA* as high-priority molecular targets. The framework subsequently classified *gyrA, gyrB, rrs,* and *eis* as medium-priority targets, with *embB, pncA, Rv0678, atpE,* and *pepQ* identified for future expansion. Importantly, these priorities were derived from the distribution of resistance categories in the surveillance dataset and established resistance mechanisms reported in the literature; mutation frequencies were not measured in the study population.

The predominance of RR-TB and MDR-TB is consistent with the continuing importance of rifampicin and multidrug resistance within DR-TB control programmes nationally and globally (6, 16, 22). WHO recommends rapid molecular testing for the detection of TB and drug resistance to facilitate timely selection of appropriate treatment (23). Rifampicin resistance is predominantly associated with mutations in *rpoB*, whereas isoniazid resistance commonly involves *katG* and the *inhA* promoter region (24). In the present dataset, RR-TB accounted for 47.5% and MDR-TB for 44.3% of cases. However, because patient-level drug-susceptibility and mutation data were unavailable, the proportion of RR-TB cases with concurrent isoniazid resistance could not be determined. Accordingly, prioritization of *rpoB, katG,* and *inhA* reflects the combined local burden of RR-TB and MDR-TB and the established molecular relevance of these targets rather than an assumption that all rifampicin-resistant isolates are also resistant to isoniazid.

The findings support consideration of local epidemiological patterns during early-stage diagnostic prioritization. Molecular assays are generally designed around established resistance mechanisms and analytical performance requirements (25, 26). Incorporating surveillance evidence may provide an additional means of identifying resistance categories of greatest local relevance and informing phased target selection (27–29). This approach is particularly pertinent where initial multiplex capacity or resources are constrained. Nevertheless, epidemiological prioritization should complement rather than replace molecular characterization, analytical evaluation, and clinical validation.

Declining treatment success and increasing proportions of unevaluated outcomes were observed among RR-TB and MDR-TB during the study period (30). These patterns indicate programme vulnerability but should not be interpreted as demonstrating that diagnostic delays or limitations caused poorer treatment outcomes. DR-TB treatment outcomes are multifactorial and may be influenced by HIV status, clinical characteristics, treatment regimens, adherence, loss to follow-up, comorbidities, socioeconomic circumstances, healthcare access, and other programme-level factors. Although diagnostic and treatment delays are recognized challenges in DR-TB care (31, 32), the aggregated surveillance data used in this study did not permit assessment of their independent contribution to the observed outcome patterns. Prospective studies incorporating patient-level diagnostic, clinical, treatment, and health-system variables are required to examine these relationships.

The prioritization of *rpoB, katG,* and *inhA* is also supported by their established biological relevance to rifampicin and isoniazid resistance. Mutations within the rifampicin resistance-determining region of *rpoB* account for a substantial proportion of rifampicin resistance, while *katG* and the *inhA* promoter are important determinants of isoniazid resistance (24, 33). These well-characterized targets therefore provide a rational starting point for future assay development. However, their prioritization in this study does not establish the local prevalence of specific mutations or their diagnostic performance in a future biosensor.

A phased approach was proposed for additional resistance-associated targets. *gyrA* and *gyrB*, together with *rrs* and *eis*, were classified as medium-priority targets because of their relevance to fluoroquinolone and selected second-line drug resistance. Additional targets, including *embB, pncA, Rv0678, atpE,* and *pepQ*, were identified for future expansion. This prioritization is intended to provide a structured approach to future multiplex development rather than a universal hierarchy of resistance markers. The clinical relevance and local frequency of specific mutations may vary geographically and over time, and mutation-level surveillance will therefore be necessary to refine the proposed target panel (34, 35).

The molecular–biophotonic component of the framework is conceptual. Optical biosensing approaches such as surface plasmon resonance (SPR), localized surface plasmon resonance (LSPR), fluorescence-based biosensing, photonic crystal sensing, and surface-enhanced Raman spectroscopy (SERS) have been investigated for nucleic acid and biomolecular detection, with published studies describing potential advantages related to analytical sensitivity, multiplexing, and miniaturization (36–38). These literature-reported characteristics were not evaluated in the present study. No biosensor was fabricated or tested, and analytical sensitivity, specificity, limit of detection, reproducibility, portability, multiplex performance, diagnostic accuracy, and clinical feasibility were not assessed. The present findings therefore support only the conceptual mapping of prioritized molecular targets to candidate technologies and should not be interpreted as validation of a molecular–biophotonic diagnostic platform.

The potential relevance of decentralized diagnostic development is particularly important in rural settings where geographic and health-system constraints may affect access to specialized laboratory services (39, 40). However, whether a molecular–biophotonic platform could reduce diagnostic delays, facilitate treatment initiation, or improve patient outcomes cannot be determined from the present data. These potential benefits require evaluation through laboratory development, implementation research, and prospective clinical studies. Such research would also need to consider cost, workflow integration, infrastructure requirements, quality assurance, acceptability, and compatibility with existing TB diagnostic pathways and WHO recommendations (41).

The principal contribution of this study is therefore the application of routine DR-TB surveillance data to an epidemiology-guided process for prioritizing molecular targets for future diagnostic research. Rather than demonstrating the performance of a diagnostic technology, the framework provides a hypothesis-generating pathway linking locally observed resistance categories with established molecular targets and candidate biosensing approaches. Its applicability beyond the study setting will require recalibration using locally representative epidemiological and molecular data.

## 5 Strengths and limitations

A strength of this study is its use of real-world DR-TB programme data from decentralized facilities serving rural communities in the Eastern Cape Province. The analysis provides a locally informed approach for identifying resistance categories that may warrant priority in future diagnostic research. The proposed framework also provides a transparent process for linking epidemiological burden with established molecular resistance targets before laboratory development. The Diagnostic Priority Index thresholds were pragmatic and study-specific rather than derived from a formally weighted or externally validated scoring system. The resulting priority categories should therefore be regarded as exploratory and may change when applied to larger, geographically diverse datasets or mutation-level surveillance data.

Several limitations should be acknowledged. First, the study relied on routine programme data collected primarily for surveillance and programme monitoring rather than diagnostic development. Consequently, potentially relevant variables, including bacterial load, diagnostic turnaround time, detailed phenotypic drug-susceptibility results, and patient-level clinical characteristics, were unavailable. Aggregated data also precluded assessment of HIV status, previous TB treatment, comorbidities, socioeconomic circumstances, adherence, treatment regimens, and other factors that may influence treatment outcomes. Therefore, the observed decline in treatment success cannot be causally attributed to diagnostic limitations.

Second, the dataset did not contain whole-genome sequencing, targeted sequencing, mutation-level line probe assay results, or other gene-level molecular data. The study could therefore not determine local mutation frequencies, characterize resistance-associated variants within individual genes, or evaluate genotype–phenotype concordance. Molecular targets were mapped from resistance categories using established resistance mechanisms reported in the literature and should therefore be interpreted as literature-informed target prioritization rather than molecular findings from the study population. Future studies should integrate phenotypic drug-susceptibility testing with targeted sequencing, whole-genome sequencing, or other validated molecular approaches to characterize local mutation patterns and refine the proposed target panel.

Third, the molecular–biophotonic component was entirely conceptual. No biosensor was fabricated, optimized, or experimentally tested, and no analytical or clinical validation was performed. Consequently, this study provides no direct evidence regarding analytical sensitivity, specificity, limit of detection, reproducibility, portability, multiplex performance, diagnostic accuracy, or operational feasibility of the proposed technologies. These characteristics require evaluation through laboratory development and prospective analytical and clinical validation.

Finally, the study was conducted at two rural facilities in the Eastern Cape Province, which may limit generalizability to settings with different resistance patterns, patient populations, and health-system characteristics. External evaluation using larger and geographically diverse datasets will be required before the prioritization framework can be considered applicable beyond the study setting.

## 7 Recommendations

Based on the findings and limitations of this study, future research should initially characterize the local mutation spectrum within the prioritized resistance categories using targeted sequencing, whole-genome sequencing, or validated molecular assays. This would enable the epidemiology-guided priorities proposed here to be refined using mutation-level evidence and would inform the selection of probes or other recognition elements for subsequent diagnostic development.

Following molecular characterization, candidate assays could initially investigate *rpoB, katG,* and *inhA*, while *gyrA, gyrB, rrs,* and *eis* could be evaluated for subsequent multiplex expansion. Additional targets, including *embB, pncA, Rv0678, atpE,* and *pepQ*, should be considered as local molecular evidence and diagnostic requirements evolve. This phased classification should remain adaptable rather than being regarded as a fixed or universally applicable target hierarchy.

Future molecular–biophotonic research should proceed through sequential laboratory development and analytical validation before clinical implementation is considered. Candidate platforms, including SPR, LSPR, fluorescence-based biosensing, and SERS, should be evaluated for analytical sensitivity, specificity, limit of detection, reproducibility, multiplex performance, stability, and operational requirements (36–38). Promising prototypes should subsequently undergo prospective clinical validation using well-characterized specimens and appropriate reference methods, including Xpert MTB/RIF Ultra, line probe assays, phenotypic drug-susceptibility testing, and sequencing approaches.

Implementation studies would then be required to determine whether validated technologies are feasible, acceptable, cost-effective, and compatible with existing rural TB diagnostic pathways. Such studies should evaluate healthcare-worker requirements, quality assurance, digital reporting, patient pathways, infrastructure, and integration with national TB surveillance and treatment programmes. Claims regarding same-day diagnosis, improved treatment initiation, reduced loss to follow-up, or improved treatment outcomes should be evaluated prospectively rather than assumed from the present surveillance analysis.

## 8 Conclusion

This study proposes an epidemiology-guided framework to prioritize molecular targets for future DR-TB diagnostic development, using routine surveillance data from the rural Eastern Cape. The predominance of RR-TB and MDR-TB supported prioritization of *rpoB, katG,* and *inhA*, with additional targets identified for phased expansion. The molecular–biophotonic component is conceptual: no mutation-level analysis, biosensor development, or analytical or clinical validation was performed. Further molecular characterization, laboratory development, analytical evaluation, and prospective clinical validation are required to determine the performance, feasibility, and potential point-of-care applicability of any resulting diagnostic platform.

## Data Availability

The raw data supporting the conclusions of this article will be made available by the authors, without undue reservation.
